# The Quarterly Review for September, 1853

**Published:** 1854-03

**Authors:** 


### ECLECTIC DEPARTMENT,

### AND SPIRIT OF THE MEDICAL PERIODICAL PRESS.

---

*The Quarterly Review for September*, 1853. Article, "Electro-Biology and Mesmerism."

We have ever opposed, to the utmost of our power, all those notions which ascribe to a new force, such as animal magnetism or tellurism, any effects on the animal body. Of the existence of such force we have no proof whatever. We also regard as unscientific and faulty the term electro-biology. By biology is understood the doctrine of life; and the great work of Treviranus on that subject should have been alone sufficient to prevent the application of a truly scientific term to a certain class of phenomena, not to speak of the improper exhibitions of perverted nervous functions which have been made in public. Such expressions as "biological," "biologized," "to biologize," etc., are altogether unmeaning and unwarrantable. Neither do we altogether approve of hypnotism introduced by Mr. Braid, for the obvious reason that sleep, in the ordinary acceptation of that word, is by no means essential for the production of the peculiar phenomena described. Still it is the best term yet proposed. As to clairvoyance, we need scarcely say, that no fact has ever come under our cognizance, which supports its occurrence, and that we utterly disbelieve in its existence, and the puerile fictions to which it has given rise. We are most anxious to impress upon our readers, our condemnation of all these expressions and theories, and this because there are certain *facts* mixed up with them, which we consider have not only been well established, but which are of a character that merit the most serious consideration of every medical man. These facts, indeed, have been constantly brought under the notice of mankind from the remotest ages, and it has simply been the difficulty of explaining them, according to the common sensations of men, and the prevailing opinions of the learned, that has given rise to all the imposture and charlatanism with which they have been connected. The article published in the last number of the "Quarterly Review," entitled "Electro-Biology and Mesmerism," was written by Dr. Carpenter, and will, we trust, go far to separate what is true from what is false connected with this matter. We can unhesitatingly assure our medical brethren, that amongst what is true there is that which demands their especial attention.

The most important fact, and one which every physiologist who has investigated the subject has fully convinced himself of, is that about one person in twenty, after gazing intently for several minutes on any fixed object, is thrown into a peculiar mental condition, closely resembling somnambulism. In this condition, by suggesting to him certain ideas, all the nervous functions may be increased, perverted or diminished. There seems to be no limit to the variety and degree in which this may be effected. The person for the time being, is governed by a dominant idea, and thinks, acts, and feels according to its dictates, in exactly the same manner as a monomaniac does. Former pains are unfelt, or new ones produced; incapacity for exertion is overcome, and the muscular strength increased, or it is rendered irregular and powerless; all the sensations may, in like manner, be influenced, and the mental processes so changed, that even self-identity may be lost, and a woman be made to think herself a man, and talk and act as she has seen him do. To enter, as Dr. Carpenter and others have done, into a long description of these particular acts and perverted sensations is unnecessary, and we have no space for it. We are told, however, that he has investigated them, and is satisfied of their reality. In 1851 they were fully investigated by Professor Bennett, and the results published by him in a pamphlet.* Sir Henry Holland, of London, in his Medical Notes and Reflections, alludes to them, and admits them as well-known occurrences. Their accuracy has been tested by numerous parties of scientific men in Edinburgh; and on one occasion the most sensitive subjects were found, among the more advanced medical students at a meeting of the Royal Medical Society, when the subject was discussed there; and at another time, they were admitted to be true, and to be within the cognizance of the members of the Medico-Chirurgical Society, at a very crowded meeting. We hold, then, that all the facts which have ever been proved to exist, or are capable of demonstration by the mesmerists, have been fully admitted and recognized by the medical profession, and been conclusively shown not to result from the influence of an external force, or of the will of another, but upon the mind or imagination of the individual affected through the influence of suggestive ideas. The recognition of the facts, and the generalization of the law which governed them, constitute a very important step in nervous physiology, for which the scientific world are undoubtedly indebted to Mr. Braid, of Manchester. On this point, Dr. Carpenter says:

"The first important step was made by Mr. Braid, a surgeon in Manchester, who discovered, about twelve years since, that a state of coma passing into somnambulism (to which he gave the appropriate designation of *Hypnotism,*) can be induced in numerous individuals of all ranks, ages, and temperaments; and that the phenomena of this state are so essentially the same with those of the (so-called) Mesmeric somnambulism, as to afford the most valuable assistance in the analysis of the real nature of the latter. In both, the somnambulist appears to be incapable of controlling his ideas, his feelings or his actions; and is entirely amenable to the will of another, who may govern the course of his thoughts at his own pleasure, and oblige him to execute

---

* The Mesmeric Mania of 1851. Edinburgh: Sutherland & Knox.

any command. The clue to the marvel was soon found by Mr. Braid, in the concentrated operation of that principle of *suggestion* which has long been known to psychologists; and under the guidance of this idea, he has subsequently followed up the investigation with great intelligence, making no mystery of his proceedings, but courting investigation in every possible way."—p. 503.

Now, in point of fact, not only was the first important step taken by Mr. Braid, but the whole subject has been thoroughly worked out by him; for to the great generalization that these phenomena are due to suggestive ideas communicated to the mind, little has been added by Professor Bennett, Sir Henry Holland, or Dr. Carpenter. All these authors, however, have given new facts and illustrations, and have pointed out how much of our every-day thoughts, functions, and acts, are attributable to suggestions derived from without. Thus, to quote from Dr. Carpenter's article:

"No one can be ignorant of the fact, that we frequently experience sensations, which, originating in our own sensorium, instead of being called forth by impressions made by external objects upon their appropriate organs of sense, are designated as *subjective.* The ringing in the ears, the flashes of light before the eyes, the nauseous tastes or disagreeable odors constantly perverting the true savor of everything that is tasted or smelled, the feeling of cutaneous irritation excited by the simple mention of the unclean torments of our beds, are familiar examples. We may cite, as parallel phenomena, those renewals of past sensations, which are often excited, with all the vividness they could derive from the actual presence of the object, by the mere forçe of mental association. Thus, it is by no means uncommon for those who suffer acutely from sea-sickness, to experience nausea at the mere sight of an agitated ocean, especially if a wave-tossed vessel be within view; and a like feeling, we are assured, has been produced by the sight of a toy, in which the motion of a ship was imitated with peculiar fidelity. We have even known a case in which a lady, who witnessed the departure of a friend by sea on a stormy day, was affected with an actual paroxysm of sea-sickness. Such facts are so familiar as to have become proverbial; for the common phrase, 'it makes me sick to think of it,' is nothing else than the expression of a physical feeling excited by mental association. There are few persons, indeed, who have not experienced the vivid return of past sensations, pleasurable or painful, when the appropriate mental state had been renewed. A Roman Catholic, who had gone to confession for the first time, when a boy, with his mouth full of the taste of a particular kind of sweet cake in which he had been indulging rather immoderately, never went on the same errand for a dozen years or more, without the distinct recurrence of the same flavor.

"Most persons have heard of the exclamation of Dr. Pearson—'Bless me, how heavy it is!' when he first poised upon his finger the globule of potassium produced by the battery of Sir H. Davy; his preconception of the association between metallic luster and high specific gravity, leading him to attribute to this new body a character which the test of the balance determined to be the opposite of the fact. So Professor Bennett mentions a case of supposed child-murder in Scotland, in which, when the coffin was exhumed, the procurator-fiscal, who attended with the medical men to examine the body, declared that he already perceived the odor of decomposition which made him feel faint, and withdrew in consequence; yet, on opening the coffin it was found to be empty; and it afterward turned out that no child had been born, and consequently no murder committed. Another case, related by Prof. Bennett, upon an authority which we know to be trustworthy, is yet more remarkable, as showing, beyond a doubt, the reality and intensity of pains, which had their origin in a mental delusion, and not in a physical lesion. A butcher, who had a shop in the market-place at Edinburgh, in trying to hang up a heavy piece of meat upon a hook above his head, lost his footing in such a manner that his arm was caught upon the hook. On being taken down and carried into the house of a neighboring surgeon, he expressed himself as laboring under the most acute agony; and the paleness of his countenance, and the almost entire absence of pulse at the wrist, were unmistakable evidences of the reality of his torture. His arm could not be moved without causing excessive pain, and he frequently cried out while the sleeve of his coat was being cut off; yet when the arm was exposed, it was found quite uninjured, the hook having only penetrated the cloth of the sleeve, and the skin being scarcely even grazed!"—p. 516–18.

Again:

"An extraordinary degree of power may be thrown into any set of muscles, by telling the somnambulist that the action which he is called upon to perform is one which he can accomplish with the greatest facility. One of Mr. Braid's hypnotized subjects—a man so remarkable for the poverty of his physical development, that he had not for many years ventured to lift a weight of twenty pounds—took up a quarter of a hundred weight upon his little finger, and swung it round his head with the utmost ease, upon being assured that it was as light as a feather. On another occasion he lifted a half hundred weight as high as the knee on the last joint of his forefinger. The impossibility of any trickery would be evident to an observant eye, since, if he had been trained to such feats (which few of the strongest men could accomplish without practice) the effect would have been visible in his muscular development. Consequently, when the same individual afterward declared himself unable to lift a handkerchief from the table, which he had been assured that he could not move, we saw no reason for questioning the truth of his conviction—based as this was upon the same kind of suggestion as that by which he had been just before prompted to a far more astonishing action." —p. 530.

Now, without citing any more passages, we would ask whether there is anything unreasonable or improbable in supposing that such an influence over the nervous functions, if judiciously exercised, may cure some diseases that have hitherto baffled the therapeutics of medical men? What practitioner of experience has not encountered hysterical females, who could not or would not walk, in whom various kinds of paralysis, convulsions, neuralgic pains, amenorrhœa, constipation, and a host of disorders have existed, which have bid defiance to every kind of drug and every kind of treatment; and yet who, on the occurrence of some mental excitement, some change in their domestic circumstances, or state of life, have suddenly become well? Is it too much to believe that functional disorders may be lessened or removed by suggestive ideas communicated to the minds of such persons, or that various secretions which are well known to be powerfully influenced naturally by the mind, may not, in like manner, be so governed by rules of art? To us this not only appears probable, but actually certain. In fact, not only are we indebted to Mr. Braid for a discovery of the law which regulates these nervous aberrations, but for its practical application to the relief and cure of diseases.

Here we quote again from Dr. Carpenter's article:

"It is found that the pulsations of the heart and the respiratory movements may be accelerated or retarded; and various secretions altered both in quantity and quality. A lady who was leaving off nursing from defect of milk, was hypnotized by Mr. Braid, and while she was in this state, he made passes over the right breast to call her attention to it. In a few moments her gestures showed that she dreamt that the baby was sucking, and in two minutes the breast was distended with milk, at which she expressed, when awakened, the greatest surprise. The flow of milk from that side continued abundant, and, to restore symmetry to her figure, Mr. Braid subsequently produced the same change on the other side; after which she had a copious supply for nine months. We are satisfied that, if applied with discrimination, the process will take rank as one of the most potent methods of treatment, and Mr. Braid's recent Essay on Hypnotic Therapeutics seems to us to deserve the attentive consideration of the medical profession."—p. 532.

The Essay on Hypnotic Therapeutics here so highly spoken of by Dr. Carpenter, was published in the last July number of this journal. We are aware that it excited considerable surprise, and difference of opinion, among some of our readers. But let us beg those who have not minutely considered this subject, to reflect, 1st, on the undoubted fact that certain persons are and can be made slaves of dominant ideas; and, 2d, on the equally undoubted fact that such mental ideas are known, by universal experience, to exercise a stimulant or depressing effect on all the bodily functions. If these propositions be admitted, what is there physiologically more extraordinary in bringing on a secretion of milk by suggesting the idea of suckling, than of causing a man's mouth to water by "bare imagination of a feast?" Is it not known that hypochondriacs, bed-ridden for years, have run with alacrity to escape from a supposed fire? and why may not an hysterical paralytic, in like manner, also walk, when the mind is stimulated by confidently telling her that she can and must do so? Is not pain always alleviated by directing the attention from the part complained of; why may not rheumatic and gouty persons be benefited by fixing their thoughts upon something else than their own ailments? The habit of going to the water-closet at a particular hour, is well known to be the best means of securing a daily alvine evacuation: why may not the command to go there, when the patient is in a certain mental state, produce relief from a temporary constipation? In short, we maintain, that so far are the statements made in Mr. Braid's paper from being incredible, that to the physiologist, and especially to one who has investigated the extraordinary power of dominant ideas, they bear inherent proofs of their correctness.

Far be it from us to check or oppose that rational skepticism in medicine which is one of its best safeguards from a too rapid revulsion, or to shorten one moment that period which is necessary to give authenticity and exactitude to alledged improvements or new facts. But this end once attained, and the alledged facts proved to be real by sober-minded men, whose interests are in no way concerned with their adoption, we are no longer warranted in ridiculing or despising them. The facts, principles, and mode of treatment brought forward by Mr. Braid have now been long tested, and are beginning to attract the attention of the profession:—and when we perceive a man of science, like Dr. Carpenter, telling us that he has personally investigated them; expressing his convictions of their truth, and endeavoring by their means to disabuse the educated public, in the pages of the "Quarterly Review," of the popular superstitions which have hitherto been mixed up with them,—it is surely incumbent on medical practitioners to be as conversant with the subject as the well-informed patients they may have to converse with. Above all, it is their professional duty to relieve pain and cure diseases, and if it can be shown that this or that method of treatment can effect the one and accomplish the other, they are bound to give it a trial. For ourselves, we feel satisfied that a more careful examination of the influence of the mind upon the body, and more especially a close observation of the effects of dominant ideas, will clear away much of the confusion and uncertainty connected with our art, and free therapeutics from a large portion of that empiricism with which hitherto it has been connected.

After what we have said of Mr. Braid, he cannot consider us insensible to his just claims in this matter. He will, therefore, we trust, excuse us for saying that much of the opposition he has experienced has arisen from the egotistical style of his writings, and the continual repetition of I did this, or I did that, and of MY process, or my treatment. Many have looked with suspicion on the small size of his publications, in 12mo or 18mo, and have thought that they were reprints from the columns of provincial newspapers. We would hint to Mr. Braid, that it is at all times difficult to awaken the attention of a practical profession like ours to new modes of thought, and especially to new kinds of treatment, and that should he be subjected to misrepresentation or calumny, the best way of meeting it is by a calm representation of authentic facts, and by his neglecting no opportunity of convincing, by positive evidence, respectable men of independent thought. We can now congratulate him on at least having secured some attention to his views, and if he would abandon the newspaper press, arrange his facts, and publish a plain, unexaggerated, and condensed account of his researches in a readable 8vo volume;—in short, if he will hypnotize himself and act on the suggestive idea we thus give him, it may happen that he will be remembered by posterity as one who has usefully contributed to the science and practice of medicine.—*Monthly Journal of Med. Science—Edinburgh.*

